# Self-organizing neural network for reproducing human postural mode alternation through deep reinforcement learning

**DOI:** 10.1038/s41598-023-35886-y

**Published:** 2023-06-02

**Authors:** Keli Shen, Guanda Li, Ahmed Chemori, Mitsuhiro Hayashibe

**Affiliations:** 1 Department of Robotics, Graduate School of Engineering, Tohoku University, Sendai, Japan; 2 LIRMM, University of Montpellier, CNRS, Montpellier, France

**Keywords:** Computational models, Machine learning, Biomedical engineering

## Abstract

A self-organized phenomenon in postural coordination is essential for understanding the auto-switching mechanism of in-phase and anti-phase postural coordination modes during standing and related supra-postural activities. Previously, a model-based approach was proposed to reproduce such self-organized phenomenon. However, if we set this problem including the process of how we establish the internal predictive model in our central nervous system, the learning process is critical to be considered for establishing a neural network for managing adaptive postural control. Particularly when body characteristics may change due to growth or aging or are initially unknown for infants, a learning capability can improve the hyper-adaptivity of human motor control for maintaining postural stability and saving energy in daily living. This study attempted to generate a self-organizing neural network that can adaptively coordinate the postural mode without assuming a prior body model regarding body dynamics and kinematics. Postural coordination modes are reproduced in head-target tracking tasks through a deep reinforcement learning algorithm. The transitions between the postural coordination types, i.e. in-phase and anti-phase coordination modes, could be reproduced by changing the task condition of the head tracking target, by changing the frequencies of the moving target. These modes are considered emergent phenomena existing in human head tracking tasks. Various evaluation indices, such as correlation, and relative phase of hip and ankle joint, are analyzed to verify the self-organizing neural network performance to produce the postural coordination transition between the in-phase and anti-phase modes. In addition, after learning, the neural network can also adapt to continuous task condition changes and even to unlearned body mass conditions keeping consistent in-phase and anti-phase mode alternation.

## Introduction

Understanding human postural coordination is essential to design rehabilitation exercises to improve the life quality of the elderly and persons with motor impairments or with brain disorders such as stroke. Exploring adaptive and synergetic posture control mechanisms is important for promoting balance recovery through effective physical training design.

Human balance control studies have been widely conducted to understand human motor learning skills in daily standing tasks^1,2^. Basic human balance control strategies such as ankle strategy, and hip strategy have been well studied through human experiments^3–5^ and computational modeling simulations^6–10^. These strategies, including the kinematic and kinetic constraints of body motors, would help us to understand the existing functional mode switch of postural coordination in balance control under various external perturbations or in related supra-postural activities.

Postural control patterns over hip and ankle joints^3^ and the relative phases, such as in-phase and anti-phase, have been defined for assessing coordination patterns. Their strengths and limitations were discussed in a study^11^ for hip-ankle postural coordination in various reaching tasks. As motion coordination units^12^, movements between the hip and ankle joints are necessary for postural control in stance^13^. The coordination of ankle and hip during the quiet standing strategy^14^, for two conditions: eyes open and close, was observed with the corresponding phase portraits of the angular displacement, velocity, and acceleration of the hip and ankle. The contributions of the ankle and hip joints were distinguished for multi-segment control in human experiments on healthy and Parkinson’s participants as well as computational model simulations^15^. Similarly, inter-segment movement coordination between hip and ankle joints influenced by the sway frequencies with regard to balance control strategies, such as ankle and hip-ankle strategy, were utilized to assess the sensory and motor impairments in incomplete spinal cord injury (iSCI)^16^. Regarding the self-organizing phenomenon of postural coordination, Bardy group works are well known that the self-organizing mode naturally emerges with two modes, “in-phase or anti-phase”, of hip and ankle joints in the human head-tracking experiments^17–19^. Depending on the target frequency in the given task to the subject, the in-phase mode was employed for low frequencies and the anti-phase mode was employed for high frequencies.

It is worth noting that optimization algorithms can be applied to implementing human-like postural motion reproduction. For instance, the transition between in-phase and anti-phase postural coordination modes was predicted by the dynamic optimization model in the previous studies^20,21^, and the influence of constraints on the selection of posture strategies was evaluated for understanding the ability of postural control. A constrained optimization process, based on the minimization of an energetic criterion with an equilibrium constraint, was proposed^21^ but this model only considered steady-state behavior. The transient dynamical behavior could be reproduced^20^ with Jacobian pseudoinverse to provide a minimum norm solution. However, the above models require body kinematics or dynamics knowledge for the model optimization approach.

However, to the best of our knowledge, there is no literature about a model-free approach that can reproduce self-organized postural coordination modes reproduction for head-tracking tasks. Particularly when body characteristics may change due to growth or aging or are initially unknown for infants, a learning capability can be essential for the hyper-adaptivity of human postural control. Thus, in this study, we propose and verify if we can reproduce a self-organizing neural network trained with a model-free learning algorithm for the human-like ’hip-ankle’ phase transition depending on the task conditions.

Therefore, we develop the learning-based method to study emergent mechanisms of the postural coordination modes in various situations. The main contributions of our study are summarized as follows.

A) A deep reinforcement learning (DRL) application has been proposed to form neural networks reproducing self-organizing postural coordination modes during head-tracking tasks.
B) The task-based constraints, and frequencies of the target point, have been taken into account and their influence on the switch of postural coordination modes is studied.
C) Different evaluation indices, such as correlation and relative phase of ankle and hip, have been used to distinguish the postural phases during head-tracking tasks, and the transient behavior between in-phase and anti-phase was reproduced with the learned neural network by a continuous alternation of the task condition.
D) The learned neural network also demonstrated in-phase and anti-phase alternation for unlearned different upper body mass conditions for transferable capability. Anti-phase was employed for the higher upper mass conditions to manage the increased inertia effect while keeping the balance.

We chose to use deep reinforcement learning in this study because it is a suitable algorithm for investigating control solution under redundancy, and with the condition of an inaccessible body dynamics for multi-objective optimization^22^.

The next of the paper is organized as follows. “Model and methodology” section introduces the model and methodology, including simulation setting, algorithm, target and reward function, and the experiment design. The adaptive motor learning results with different experimental settings are analyzed in “Motor learning results on postural tasks” section. In “Further discussion” section, the relation between influential factors and phase coordination is further discussed. The conclusions and perspectives of this study are summarized in “Conclusion and perspective” section.

**Figure 1:**
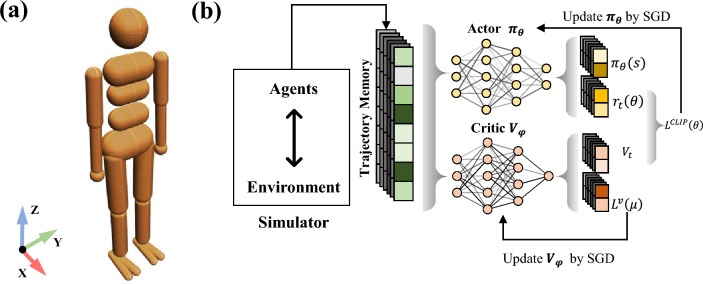
(**a**) Simulation model of a humanoid in Isaac Gym. (**b**) The training framework of PPO, which is one type of deep reinforcement learning with actor critic structure.

## Model and methodology

### Simulation setting

**Table 1:** Model parameters of a humanoid agent in Isaac Gym.

Weight	40.15 kg
Height	1.57 m
Position of CoM	[0.006, 0, 0.814] m
Constrained range of hips	(-120, 45) deg on y-axis
Constrained range of ankles	(-50, 50) deg on y-axis
Maximum output torques of joints	(-100, 100) Nm
Position of target	[x, 0, 1.7] m

Training neural networks using deep reinforcement learning requires collecting data generated during the humanoid agent’s interaction with the external physical environment under gravity. We conducted our experiments in the simulation environment with the physics engine provided by Isaac Gym^23^.

Isaac Gym is a high-performance GPU-based physics simulation engine designed for robot learning by NVIDIA company^23^. With end-to-end GPU acceleration and parallel processing of thousands of simulation environments, Isaac gym can greatly increase the training speed when performing reinforcement learning tasks compared to other simulation software environments.

The simulation model we used for the experiment is modified based on the humanoid agent in Isaac Gym, as illustrated in Fig. 1. The original version of the humanoid robot has 21 degrees of freedom. In order to exclude irrelevant variables, we kept only the hip and ankle joints on both body sides and fixed the other joints in our simulations. The distance between two feet was the shoulder width. By giving the mirrored input to the same joints, we reduce the humanoid’s range of motion to a 2D space. Then the robot has four degrees of freedom: hip and ankle for the right and left leg. The other physical parameters of the simulation model are shown in Table 1.

### Algorithm

We use Proximal Policy Optimization (PPO)^22^ to train the robot in our experiment, which is one of the state-of-the-art deep reinforcement learning algorithms based on the actor-critic framework. PPO can simultaneously collect training data from multiple agents based on a parallel architecture with synchronous updates. This allows it to take full advantage of Isaac gym parallel simulation on GPU to accurate the training process.

As shown in Fig. 1b, in the training framework of PPO, there are two neural networks, one is the actor network $\pi _{\theta }\left( a_{t} \mid s_{t}\right)$ and the other is critic network $V_{\phi }\left( s_{t}\right)$. The actor network $\pi _{\theta }$ is used to sample action $a_t$, according to the input state $s_t$ at each control timestep. The critic network $V_{\phi }\left( s_{t}\right)$ is the value function that can estimate the expected average return starting from state $s_t$.

1 $$\begin{aligned} V_{\phi }\left( s_{t}\right) ={\mathbb {E}}\left[ R_t \mid \pi _\theta ,s_t\right] , \end{aligned}$$

PPO updates the actor network and critic network based on the experience collected from the interaction between the agent and the environment. The loss function used to update the actor-network $\pi _{\theta }$ is expressed as follows:

2 $$\begin{aligned} L^{C L I P}(\theta )=\hat{\mathbb {E}}_{t}\left[ \min \left( \omega _{t}(\theta ) \hat{A}_{t}, {\text {clip}}\left( \omega _{t}(\theta ), 1-\varepsilon , 1+\varepsilon \right) \hat{A}_{t}\right) \right] , \end{aligned}$$

in which $\hat{A}_{t}$ is the estimated advantage at time *t*. The advantage $A_t$ is used to evaluate how good or bad the action $a_t$ taken in state $s_t$ is. $A_t$ greater than zero indicates that the action currently taken can get more reward than expectation and vice versa.

PPO uses a method called “clipping” to limit the magnitude of the updates, which helps improve the stability of training. The likelihood ratio $\omega _t$ is used to measure the difference between the new policy $\pi _{\theta }$ and the old policy $\pi _{\theta _{\text{ old } }}$.

3 $$\begin{aligned} \omega _{t}(\theta )=\frac{\pi _{\theta }\left( a_{t} \mid s_{t}\right) }{\pi _{\theta _{\text{ old } }}\left( a_{t} \mid s_{t}\right) }, \end{aligned}$$

If $\omega _t$ beyond the range of $\left( 1-\varepsilon , 1+\varepsilon \right)$, the clip function ${\text {clip}}\left( \omega _{t}(\theta ), 1-\varepsilon , 1+\varepsilon \right)$ will sets the gradient to zero. Then the minimum function will select a lower bound for $L^{C L I P}(\theta )$ between the clipped and unclipped advantages.

The loss function for the critic network is denoted as:

4 $$\begin{aligned} L_{t}^{V F}(\phi )=\left( V_{\phi }\left( s_{t}\right) -V_{t}^{{\text {target}}}\right) ^{2}, \end{aligned}$$

in which $V_{\theta }$ is the estimated value and $V_{t}^{{\text {target}}}$ is the real value. The hyper-parameters of PPO, used in our experiments, are listed in Table 2.

**Table 2:** Hyper-parameters for training humanoid agent by PPO.

Environments	8192
KL threshold	0.002
Mini-batch size	8192
Horizon length	32
PPO epochs	5
Hidden layers	[256, 256]
Training steps	5,000,000

### Target and reward functions

Our target is to let the humanoid’s head track a periodic reciprocal motion in the X-axis direction in the sagittal plane. We use the red ball above the humanoid’s head to indicate the location of the target point for better visualization. The motion equation of the trajectory for the head-tracking target is formulated as:

5 $$\begin{aligned} x=-A\cos (2\pi ft)+A, \end{aligned}$$

where *A* is the amplitude, such that the target point moves along the X-axis in the range [0, 2*A*]. *f* denotes the movement frequency of the target point. This target setting is identical to the human head-tracking experiment employed in the study^19^ as they performed anteroposterior directional target tracking with the head for the standing participants.

Reward function $R_{total}$ is expressed as follows:

6 $$\begin{aligned} R_{total}= & {} \alpha R_{stay}+\beta R_{target}+\gamma R_{energy}+\gamma R_{torque} \end{aligned}$$

7 $$\begin{aligned} R_{stay}= & {} (1+\Vert P_{f0}-P_{ft} \Vert _2^{2})^{-1} \end{aligned}$$

8 $$\begin{aligned} R_{target}= & {} (1+\Vert P_{target_x}-P_{head_x} \Vert _2^{2})^{-1} \end{aligned}$$

9 $$\begin{aligned} R_{energy}= & {} - \sum _{J}\left| \tau _j\omega _j \right| \Delta t \end{aligned}$$

10 $$\begin{aligned} R_{torque}= & {} - \sum _{J} (\tau _j)^2 \end{aligned}$$

where $R_{stay}$ is used to limit the movement of the humanoid’s foot relative to the ground, where $P_{f0}$ is the initial position of the humanoid foot and $P_{ft}$ is the position of the humanoid foot at time *t*. $R_{target}$ is used to encourage the humanoid to track the target, where $P_{target_x}$ is the position of the target point at the x-axis and $P_{head_x}$ is the position of the humanoid head at x-axis. $R_{energy}$ and $R_{torque}$ are used to limit the robot’s energy consumption, where $\tau _j$ is the torque of the joint *j*, $\omega _j$ is the angular velocity of the joint *j* and $\Delta t$ is the time interval between sampling simulation data. If the height of the center of mass of the humanoid torso is less than 1m, the humanoid is judged to have fallen, and $R_{total}$ will be set to -1. The weights for adjusting each reward term are the $\alpha$, $\beta$, and $\gamma$.

### Experiment design

There are many factors that may affect the humanoid’s postural motion coordination. In our experiments, we analyzed the effects of the motion frequency of the target point, the energy consumption weight in the reward of the learning process, and after learning unlearned upper body mass condition was changed to check also for a new task.

The energy consumption is adjusted by tuning the variable $\gamma$ in Eq. (6). The frequency of the target point is adjusted through the variables *f* in Eq. (5).

## Motor learning results on postural tasks

This section studies the influence of task-based constraints on the self-organization of postural modes between in-phase and anti-phase during tracking-balancing tasks. The impact of energy consumption penalization on head-tracking tasks has also been studied. Energy consumption has been verified as an essential factor for upright standing tasks in human experiments^24^ and simulations^7,20,21,25^. This is reasonable since our human body tries to save energy consumption with an energy-optimal policy when completing specific motion tasks in our daily activities.

These task constraints are considered in tracking-balancing simulations implemented through a model-free learning method, and their influence mechanism is explored in this study. The task-based constraints, and frequency of head-tracking target point, have been studied for the influence on hip and ankle coordination in human experiments^17,19,26^, and simulation of the double inverted pendulum model with feedback^20^. However, their work did not study the learning-based adaptivity of motor coordination. Therefore, verifying if model-free learning methods can establish a neural network to create adaptive self-organizing behavior, is important, to study further the effectiveness of in-phase and anti-phase coordination modes between hip and ankle joints.

Although the head tracking task is not a common activity in daily life, it is equivalent to maintaining head stability when the floor is accelerating or decelerating. For small disturbances, in-phase balancing is sufficient, but for larger disturbances, anti-phase balancing is required. This situation often occurs when we ride a bus or train in daily life. By adjusting head speed, we can alter the disturbance intensity.

**Figure 2:**
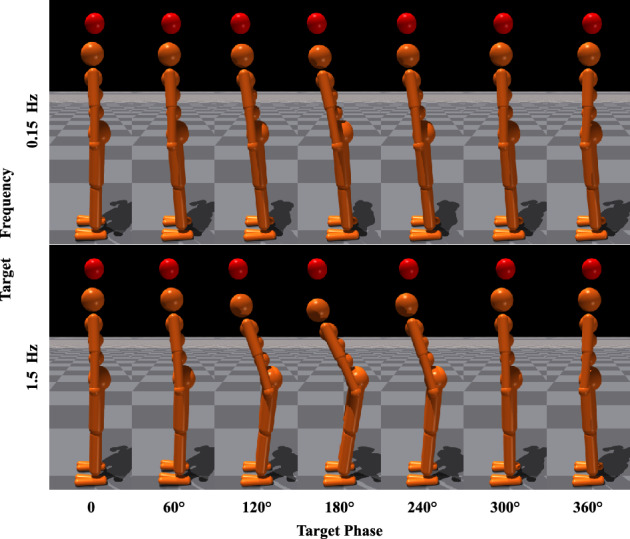
Screenshots of learning tracking-balancing tasks at motion tracking frequency *f* of target point in Eq. (5) with regard to 0.15 [Hz] and 1.5 [Hz]. Where, energy consumption penalization parameter $\gamma$ in Eq. (6) is 20. The motion amplitude *A* of target point in Eq. (5) is 0.1 [m]. Ankle and hip stiffness remains 25 [Nm/rad] and 125 [Nm/rad].

**Figure 3:**
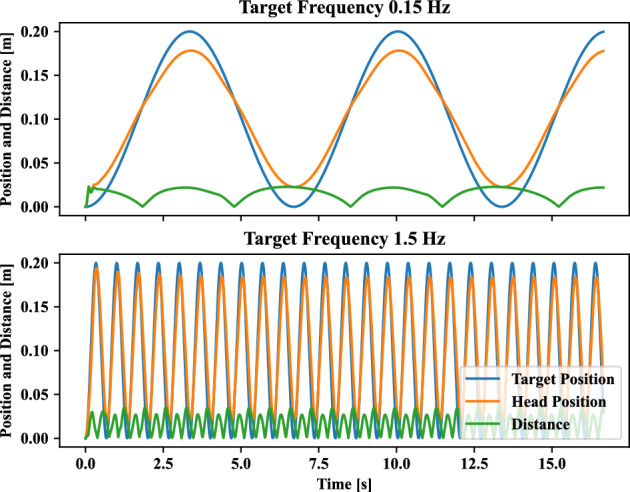
Position of tracking target and humanoid agent head and its distance at motion tracking frequency *f* in Eq. (5) with regard to 0.15 [Hz] and 1.5 [Hz]. Where, energy consumption penalization parameter γ in Eq. (6) is 20. The motion amplitude *A* of target point in Eq. (5) is 0.1[m]. Ankle and hip stiffness remains 25 [Nm/rad] and 125 [Nm/rad].

**Figure 4:**
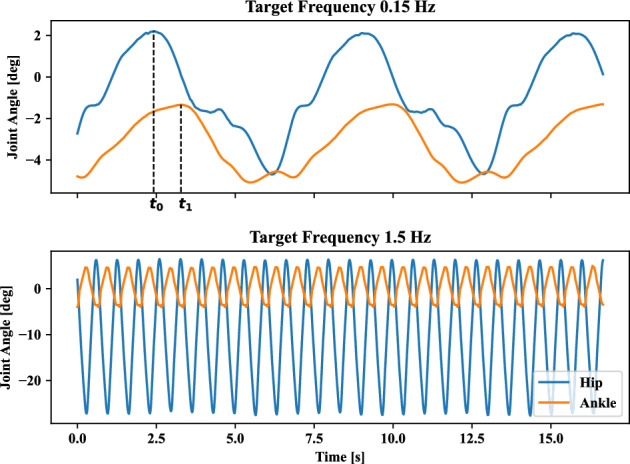
Joint angle of ankle and hip at motion tracking frequency *f* in Eq. (5) with regard to 0.15 [Hz] and 1.5 [Hz]. Where, energy consumption penalization parameter $\gamma$ in Eq. (6) is 20. The motion amplitude *A* of target point in Eq. (5) is 0.1 [m]. Ankle and hip stiffness remains 25 [Nm/rad] and 125 [Nm/rad]. $t_0$ and $t_1$ are the timesteps corresponding to the maximum angles of the ankle and hip in the current motion cycle.

### Experiment 1: effect of the frequency of tracking target

This section demonstrates how the tracking frequency affects self-organized postural coordination mode. The change in frequency of the tracking target can determine the movement speed of the head for the humanoid model to complete the target-tracking tasks. Furthermore, the motion speed change can influence the joint coordination modes keeping balance performance stability. Thus, the in-phase and anti-phase patterns can be recruited with regard to the tracking frequency. The ten different tracking frequencies are set as: 0.15,  0.30,  0.45,  0.60,  0.75,  0.90, 1.05, 1.20, 1.35 and 1.50 [*Hz*]. Screenshots of learning tracking-balancing tasks at motion tracking frequency *f* of target point in Eq. (5) with regard to 0.15 [*Hz*] and 1.5 [*Hz*] are depicted in Fig. 2. In the case that energy consumption penalization parameter $\gamma$ in Eq. (6) is 20. The motion amplitude *A* of target point in Eq. (5) is 0.1[*m*]. Ankle and hip stiffness remains 25 [Nm/rad] and 125 [Nm/rad]. From Fig. 2, the in-phase motor patterns between the ankle and hip joints in the first row are kept for the target frequency 0.15 [*Hz*], while the anti-phase motor patterns between the ankle and hip joints in the second row appear in target phase $60^{\circ }$, $120^{\circ }$, $180^{\circ }$, $240^{\circ }$ and $300^{\circ }$ for the tracking target frequency 1.5 [*Hz*]. The corresponding head motion example is shown in Fig. 3. The position of tracking target and humanoid agent head is plotted at motion tracking frequency 0.15 [*Hz*] and 1.5 [*Hz*]. The corresponding joint angles for the ankle and hip are shown in Fig. 4 where we can confirm in-phase for low frequency and anti-phase for higher frequency.

#### Performance of tracking-balancing tasks

**Table 3:** Tracking performance of different target frequency.

Freq. [Hz]	r	Amplitude [m]	Head Vel. [m/s]
0.15	0.9984	0.0781	0.0464
0.3	0.9973	0.0782	0.0915
0.45	0.9955	0.07845	0.1371
0.6	0.9934	0.07875	0.1842
0.75	0.9910	0.079	0.2320
0.9	0.9880	0.0791	0.2775
1.05	0.9839	0.0797	0.3253
1.2	0.9801	0.08035	0.3768
1.35	0.9766	0.08135	0.4259
1.5	0.9669	0.0829	0.4800

First, the corresponding tracking accuracy is quantified through the target-head correlation *R*, mean amplitude of the head movement, and mean head velocity as shown in Table 3. The target-head correlation *R* is computed by Person correlation coefficients. We can confirm that the head tracking task could have been done correctly from the high value of correlation *R* in Table 3. For the higher target motion frequency over 1.2 [*Hz*], we observe a small decrease in target-head correlation *R* since the high tracking speed may increase the difficulty of tracking tasks.

**Figure 5:**
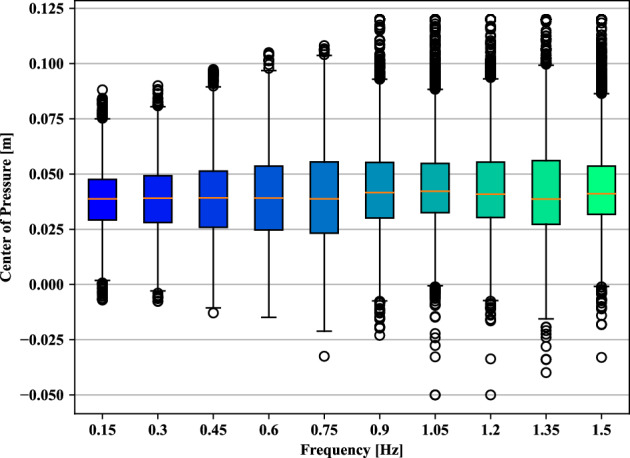
The box plot of the center of pressure (CoP) influenced by different motion tracking frequencies *f* of the target point in Eq. (5) 0.15,  0.30,  0.45,  0.60,  0.75,  0.90, 1.05, 1.20, 1.35 and 1.50 [Hz], respectively. Where, energy consumption penalization parameter $\gamma$ in Eq. (6) is 20. The motion amplitude *A* of target point in Eq. (5) is 0.1 [m]. Ankle and hip stiffness remains 25 [Nm/rad] and 125 [Nm/rad].

Second, the center of pressure (CoP) is used to estimate the balance stability. The box plot of the center of pressure (CoP) influenced by the following motion tracking frequencies *f* of the target point in Eq. (5) 0.15,  0.30,  0.45,  0.60,  0.75,  0.90, 1.05, 1.20, 1.35 and 1.50 [*Hz*], respectively are depicted in Fig. 5. The distribution of CoP increases with the increase of tracking frequency. For high-speed tracking performance, there should be a risk of losing balance. Therefore, balance maintenance is a crucial component in high-speed tracking-balancing tasks. In our modeling, the humanoid agent can finish the tracking-balancing tasks for a wide range of frequency and the feet are with unilateral constraints. This demonstrates the robustness of our proposed trained networks by PPO.

#### Emergent transition of postural modes

**Figure 6:**
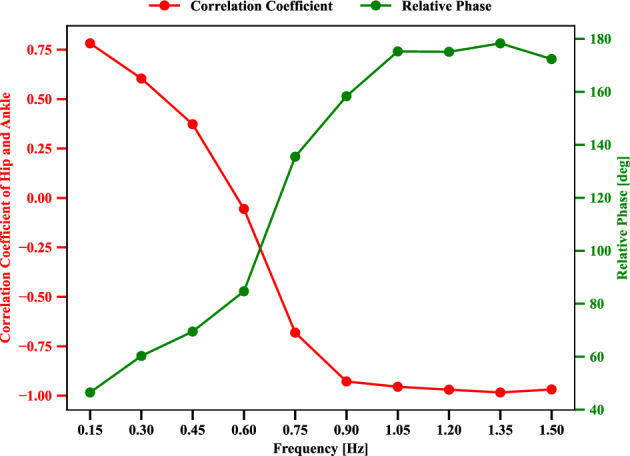
Evolution of correlation coefficient and relative phase influenced by different motion tracking frequencies *f* of the target point in Eq. (5) 0.15,  0.30,  0.45,  0.60,  0.75,  0.90, 1.05, 1.20, 1.35 and 1.50 [Hz], respectively. Where, energy consumption penalization parameter $\gamma$ in Eq. (6) is 20. The motion amplitude *A* of target point in Eq. (5) is 0.1 [m]. Ankle and hip stiffness remains 25 [Nm/rad] and 125 [Nm/rad].

Tracking frequency is an impact factor for changing the motor coordination patterns of the ankle and hip joints, such as in-phase and anti-phase for tracking-balancing tasks^27^. Therefore, we tried to understand the self-organization of postural coordination modes influenced by the tracking frequency in our tracking-balancing tasks. The evolution of correlation coefficient, relative phase influenced by the following motion tracking frequencies *f* of the target point in Eq. (5) from 0.15 to 1.50 [*Hz*], respectively, is depicted in Fig. 6. Where, energy consumption penalization parameter $\gamma$ in Eq. (6) is 20. The motion amplitude *A* of target point in Eq. (5) is 0.1[*m*]. Ankle and hip stiffness remains 25 [Nm/rad] and 125 [Nm/rad]. The correlation coefficient of hip and ankle joint angles can summarize the characteristics of the whole coordination patterns. As we can observe from Fig. 6, for tracking frequencies 0.15,  0.30,  0.45, and  0.60 [*Hz*], the corresponding positive correlation means that the in-phase patterns functioned between the ankle and hip joints. However, the negative correlation for the tracking frequencies  0.75,  0.90, 1.05, 1.20, 1.35, and 1.50 [*Hz*] indicates the anti-phase patterns between the ankle and hip joints. Thus, the transition of postural coordination modes depends on the change of the tracking frequencies with regard to relatively low and high values as the above experiment setting. Furthermore, the hip and ankle correlation coefficients are negatively correlated with the relative phase.

Next, we discuss how the change in tracking frequency influences the ankle and hip joint angles. In Fig. 6, the relative phase positively correlates with the tracking frequency. Relative phase was computed as $\left| t_0-t_1\right| \times f \times \pi$, where *f* is the motion frequency of the target, $t_0$ and $t_1$ are the timesteps corresponding to the maximum angles of the ankle and hip in the current motion cycle as shown in Fig. 4. Because the high tracking frequency means fast speed tracking motion tasks. This quick response of humanoid agents may cause unsafe performance unless the ankle and hip joints increase the motion intensity in an opposite direction to compensate for the acceleration/deceleration of the upper limb for the possible falling risks. Then, there is no solution space with in-phase joint usage due to balance maintenance requirements. However, there is a solution to keep the whole-body center of mass projection to the ground, close to the foot area if the ankle and hip are coordinated in anti-phase.

**Figure 7:**
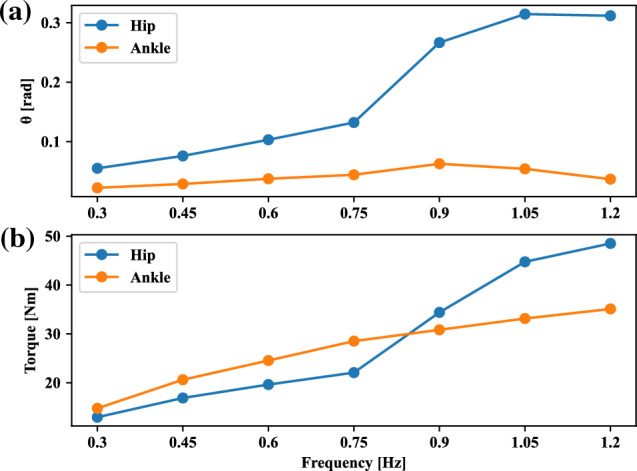
(**a**) Peak-to-peak joint positions. Each point is the mean value of simulation results in 100 s. (**b**) Torque amplitudes of ankle and hip. Hip torque is smaller for in-phase and larger for anti-phase.

The averaged peak-to-peak joint positions for the hip and ankle are plotted in Fig. 7 to show how the hip joint range was changed from low frequency to high frequency. We can observe the increase of hip amplitude along with the hip torque increase while having a similar motion range for the ankle joint. This trend is in agreement with the previous human measurement^19^ and also with previous model-based simulation results^20^. It is good to confirm the same self-organizing process could be reproduced with the learned neural network without prior dynamics and kinematics information usage. Since the range of motion considering angular positions of hip joints are much larger than those of ankle joints for high frequency tracking, we can understand that hip joints play the active and leading coordination for high-speed tracking movements. It is worth noting that the tracking frequency in the given task can determine how to employ the coordination modes of the ankle and hip joints.

The study of balance control with different head tracking frequencies, and its analysis with different motor coordination modes has significant implications for clinical applications. Understanding how head tracking frequency affects motor coordination modes can inform the development of effective rehabilitation programs for individuals with balance impairments, such as those with neurological conditions. By designing rehabilitation programs that target specific motor coordination modes, we can improve postural control and reduce the risk of falls. Moreover, studying motor coordination modes can help identify individuals who may be at a higher risk of falls due to altered coordination patterns. By examining motor coordination modes and their relationship to head tracking speeds, clinicians can develop targeted interventions to improve postural control and reduce fall risk in high-risk populations, such as older adults or individuals with neurological conditions.

### Experiment 2: effect of the energy penalization of agent

After studying the postural mode transitions by task constraints, let us now focus on another factor, energy penalization in the reward. Energy consumption can determine which type of motion policy is to be applied for implementing motion tasks. Therefore, we assume that energy consumption might change the postural coordination between the ankle and hip joints. From Eq. (6), $R_{energy}$ and $R_{torque}$ are used to limit the robot’s energy consumption. In this work, to understand the influence of energy penalization on the postural coordination during tracking-balancing tasks, we tune the energy weight (energy index) $\gamma$ in Eq. (6) with the following values: 0, 5, 10, 15, 20, 25, 30, 35, and 40. Then, the performance of tracking-balancing tasks and the transition of postural coordination are studied separately.

#### Performance of tracking-balancing tasks

**Table 4:** Tracking performance of different energy index.

Energy index $\gamma$	r	Head Amp. [m]	Energy cost [J]
0	0.9935	0.0900	1632.3
5	0.9984	0.0982	498.6
10	0.9826	0.0945	379.8
15	0.9836	0.0955	340.8
20	0.9798	0.0840	287.3
25	0.9951	0.0989	381.0
30	0.9909	0.0959	322.1
35	0.9979	0.1000	346.9
40	0.9979	0.1000	340.4

Target-head correlation *R* could be kept high for each energy index according to the results of Table 4. These correlations show the tasks were properly performed for head tracking tasks even for different settings of energy penalty. The corresponding head amplitude also shows a similar head motion tendency regardless of the energy penalty. Thus, we can assume that energy-saving can be done without affecting the head tracking accuracy itself. Table 4 also demonstrates that the required energy cost could be well decreased by following the energy penalty. Thus, the motion could be created with lower energy while keeping the task performance.

#### Emergent transition of postural modes

**Figure 8:**
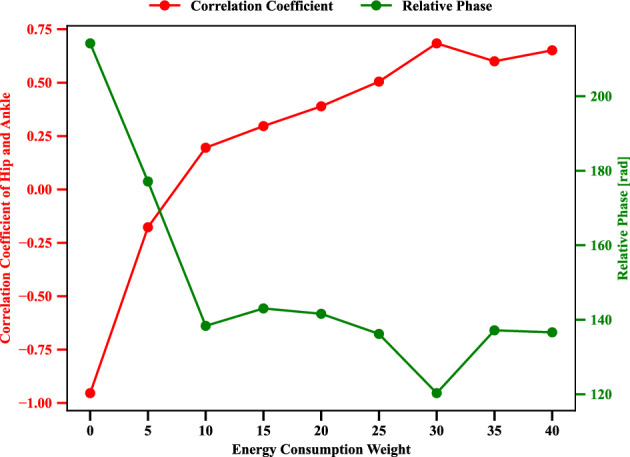
Evolution of correlation coefficient, relative phase influenced by different energy consumption penalization parameter $\gamma ~=$ 0, 5, 10, 15, 20, 25, 30, 35 and 40 in Eq. (6), respectively. Where the motion amplitude *A* of target point in Eq. (5) is 0.1 [m]. Ankle and hip stiffness remains 25 [Nm/rad] and 125 [Nm/rad]. And the motion tracking frequency *f* of the target point in Eq. (5) is 0.4 [Hz].

In this scenario, the postural coordination between the ankle and hip joints is studied by tuning the energy weight in the training reward function. The correlation coefficient and relative phase are recruited to quantify the transition of coordination modes. The evolution of the correlation coefficient, relative phase influenced by different energy penalization parameters $\gamma ~=$ 0,  5,  10,  15,  20,  25,  30, 35 and  40 in Eq. (6), respectively, are displayed in Fig. 8. Where the motion amplitude *A* of target point in Eq. (5) is 0.1[*m*]. Ankle and hip stiffness remains 25 [Nm/rad] and 125 [Nm/rad]. And the motion tracking frequency *f* of target point in Eq. (5) is 0.4 [*Hz*].

With the increase in energy weight, the hip and ankle correlation coefficient changes from a negative value to a positive one. The negative correlation coefficient for the cases of energy weight $\gamma ~=$ 0 and  5 in Eq. (6) expresses the anti-phase modes between the ankle and hip joints. However, the in-phase modes between the ankle and hip joints appear for large energy weight $\gamma ~=$ 10, 15, 20, 25, 30, 35 and 40 in Eq. (6), respectively. From an energy-saving point of view, in-phase coordination consumes less energy than anti-phase coordination between the ankle and hip joints if there is a solution to manage the given balancing and head tracking task. Then, in this case, it can be considered there were two types of solutions by in-phase and anti-phase, but by employing energy penalty, energy inefficient solution could be removed then it has resulted in an in-phase solution. Anti-phase modes enhance adaptive motor flexibility by consuming more energy during tracking-balancing tasks even when there is no solution with in-phase modes. However, when both in-phase and anti-phase could be available, it is better to remove the anti-phase solution by employing an energy penalty. This is understandable, considering the relationship between energy consumption and task management.

Furthermore, the relative phase decreases with the increase in energy consumption weight. This indicates that energy consumption modification for tracking-balancing tasks may change the kinematic features of motor coordination between the ankle and hip joints. As discussed above, the motion modes switch from anti-phase to in-phase to pursue energy-saving goals during managing the given task. Additionally, we can conclude that energy penalization tends to change the ankle and hip joints’ coordination modes toward in-phase energy efficient motor mode in principle, however, the energy weight setting is not highly sensitive for the postural mode selection as we can observe stable relative phase relationship after having more than 10 value for the weight. The energy penalty weight in the reward is a hyper-parameter in the learning process. We could confirm that we need some energy penalty consideration in the reward, however, it is not a highly sensitive factor for reproducing the consistent in-phase and anti-phase mode employment.

### Experiment 3: changing the hip stiffness of agent

Previous studies show that human joint stiffness differs mainly by age^28,29^, body size^30,31^ and sex^32–36^. Joint stiffness is an essential factor for balance control in quiet standing^28^, and it increases with age in postural control^29^. The change in hip stiffness may influence the range of motion since hip stiffness changes its recovery torque to return to the hip joint’s neutral position. For instance, the recent study^37^ indicates that relationships between a maximum range of motion and hip stiffness during quiet standing are consistent at common angles. Thus, the increase in hip stiffness may change the postural coordination of the hip and ankle during tracking-balancing tasks. In this study, we propose to change the humanoid model’s left and right hip stiffness together. The eight conditions are given here based on the biomechanical standards: 25,  50,  75,  100,  125,  150,  175, and  200 [*Nm*/*rad*]^20,38–40^.

**Figure 9:**
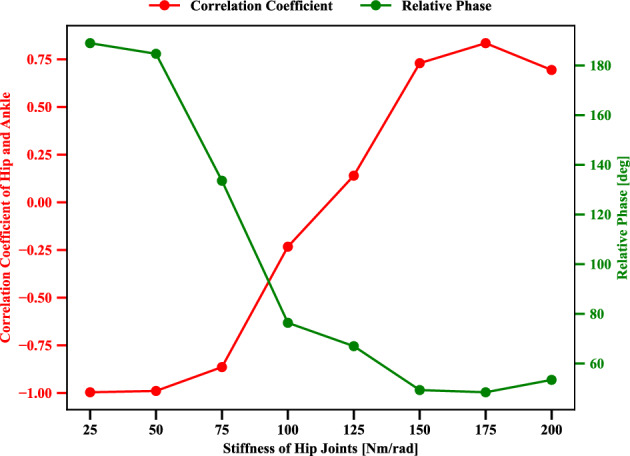
Evolution of the correlation coefficient, relative phase influenced by different hip stiffness values 25,  50,  75,  100,  125,  150,  175, and 200 [Nm/rad], respectively. Energy consumption penalization parameter $\gamma$ in Eq. (6) is 20. The motion amplitude *A* of target point in Eq. (5) is 0.1 [m]. Ankle stiffness remains 25 [Nm/rad]. And the motion tracking frequency *f* of target point in Eq. (5) is 0.5 [Hz].

In this scenario, we try to understand the influence of different hip stiffness on postural motor coordination. As mentioned above, stiffness can be an important mechanical constraint that may change the motion modes. Two evaluation indices, such as correlation coefficient and relative phase, have been proposed to study the switch of motion modes from in-phase to anti-phase between hip and ankle. The evolution of correlation coefficient, relative phase influenced by different hip stiffness values 25,  50,  75,  100,  125,  150,  175, and  200 [*Nm*/*rad*], respectively are shown in Fig. 9. Where energy consumption penalization parameter $\gamma$ in Eq. (6) is 20. The motion amplitude *A* of target point in Eq. (5) is 0.1[*m*]. Ankle stiffness remains 25 [Nm/rad]. And the motion tracking frequency *f* of target point in Eq. (5) is 0.5 [*Hz*].

As the increase of hip stiffness, the correlation coefficient of the hip and ankle changes from a negative value to a positive one. For small stiffness values, such as 25,  50,  75, and  100 [Nm/rad], the corresponding negative correlation expresses the anti-phase coordination between the ankle and hip during the tracking-balancing tasks. However, a positive correlation with regard to the hip stiffness  125, 150, 175, and  200 [Nm/rad] demonstrates the in-phase postural modes between the ankle and hip during tracking-balancing tasks. For the cases with higher stiffness, there is supporting torque to return to its neutral position, then the upper body inertia effect during the head tracking task appears relatively smaller then in-phase mode can be still employed. For frequency-increased cases, anti-phase mode gets necessary for managing the higher body inertia effect. In the case of higher stiffness, the inertia effect is decreased then an in-phase solution can be still used. Instead, when the joint stiffness gets decreased, the necessity to compensate for the body inertia gets high, then anti-phase mode coordination is more required. This is reasonable since anti-phase motor coordination for small stiffness needs to recruit large motion intensity. In contrast, in-phase motor coordination for large stiffness recruits small ankle and hip motion intensity. The corresponding green curve shows that as the hip stiffness increases, the relative phase decreases, which has been observed from human experiments^17,19^.

Individuals with certain musculoskeletal conditions or injuries may experience changes in joint stiffness that affect specific joints during clinical rehabilitation. For example, individuals with hip osteoarthritis may experience increased hip stiffness due to changes in the articular cartilage or joint capsule, while their ankle joint stiffness may remain unchanged. Similarly, individuals who have undergone hip replacement surgery may experience changes in hip stiffness. The self-organizing postural control system, which involves the recruitment of hip-ankle coordination modes such as in-phase and anti-phase, could help understand better standing ability in the daily life of these individuals.

### Experiment 4: new task adaptability with mode alternation

#### Continuous variation of frequency

In the previous sections, we could form a self-organizing neural network that employs mode alternation depending on the given tasks or the conditions. For the learning process, there was the information and the experience for the given frequencies. In addition, the neural network is trained under different frequencies but they were given as the fixed setting, not with continuous variation of the frequency.

**Figure 10:**
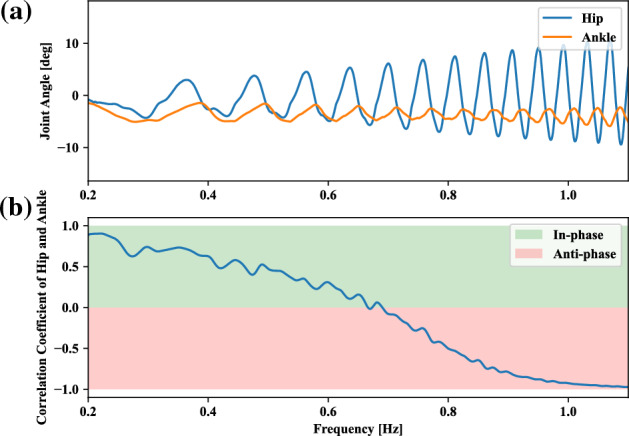
Typical result for continuous target frequency variation. (**a**) Joint angle variation of ankle and hip. (**b**) The correlation coefficients between the ankle and hip joints with increasing target frequency. The transition frequency is 0.69 Hz. The window length for calculating the correlation coefficient is 300 timesteps. The growth rate of the frequency is 0.036 Hz/s. The simulation time is 25 s.

In this section, against the learned neural network model, the behavior under the continuous variation of frequency is studied regarding the postural coordination of ankle and hip joints. In-phase between hip and ankle joint was employed for the low frequency, With the increase of target frequency, the mode was gradually shifted into anti-phase as shown in Fig. 10, where the frequency was increased linearly over time. The growth rate of the frequency is 0.036 Hz/s for 25 s. Fig. 10 reveals a transient change in coordination mode even for the continuous frequency, even when the neural network is trained only with the fixed different settings. It indicates that the neural network has certain transferable capability even for the new task which is given firstly if the frequency range is within the experienced scale. The generated motion in experiment 4 can be confirmed in the video (Supplementary file 1).

#### New task with upper body mass increase

By increasing frequency during the head tracking task, the target velocity changes with more large scale, it naturally involves larger mass acceleration and deceleration of the upper body. Larger mass acceleration and deceleration of the upper body can happen also for the increased upper body mass even for the normal target frequency. Expecting the learned neural network to manage the larger inertia effect, we have verified how the neural network may behave for the new task with an upper body mass increase. It is worth noting that the neural network is trained only with different target frequencies as described in the section of Experiment 1.

**Figure 11:**
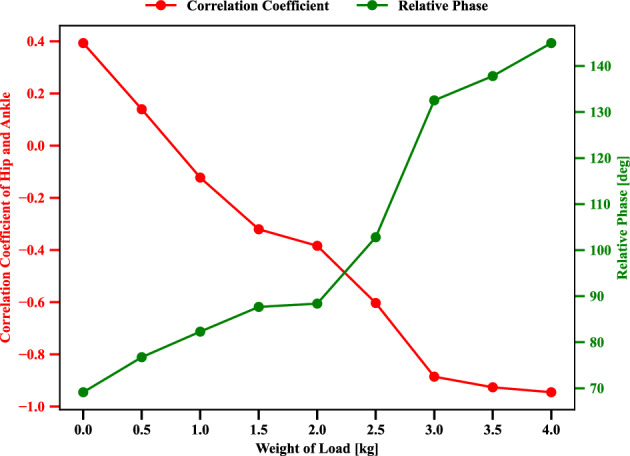
Evolution of correlation coefficient, relative phase influenced by the different weight of the added load at the upper body. Where energy consumption penalization parameter $\gamma$ in Eq. (6) is 20. Ankle and hip stiffness remains 25 [Nm/rad] and 125 [Nm/rad]. The motion tracking frequency *f* of target point in Eq. (5) is 0.4 [Hz].

**Figure 12:**
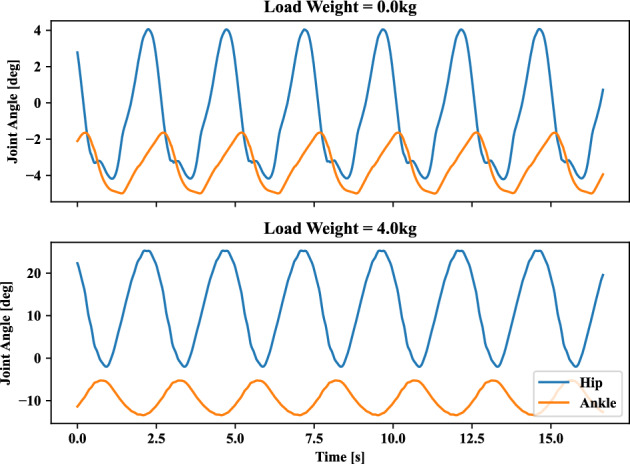
Joint angle of the ankle and hip in the different weight of the load. Where energy consumption penalization parameter $\gamma$ in Eq. (6) is 20. Ankle and hip stiffness remains 25 [Nm/rad] and 125 [Nm/rad]. The motion tracking frequency *f* of target in Eq. (5) is 0.4 [Hz].

This scenario deals with the transfer learning capability of the self-organizing neural network on the postural coordination of ankle and hip joints, and it was evaluated through the corresponding correlation coefficient and relative phase. These indices can illustrate the transition of emergent modes and kinematic features, as discussed in Experiment 1. The evolution of the correlation coefficient and relative phase influenced by different added upper body mass from 0.0 to 4.0 [*kg*], respectively, are illustrated in Fig. 11. The motion tracking frequency *f* of target in Eq. (5) is 0.4 [*Hz*]. At this target frequency, as it is relatively low frequency, the in-phase mode is normally employed as in Experiment 1 as can be confirmed in the upper plot in Fig. 12. While the added upper body increases from 0.0 to 4.0 [*kg*], the correlation coefficient of the hip and ankle decreased from positive to negative. This means that the postural coordination modes turned into switching from in-phase to anti-phase. It is interesting to observe this self-organizing mode alternation even when this task of increased upper body mass is newly given to the neural network.

Many people move with backpacks to work or school as daily activity, the weight of these backpacks varies from day to day. Carrying a backpack can often lead to an increase in upper body mass, which can challenge balance control and postural stability. This is especially true when the weight of the backpack is unknown beforehand. As such, we included this scenario in the study to examine the effects of carrying a load on the back on balance control and postural stability with a focus on hip-ankle motor coordination mode. For example, individuals with hip or ankle injuries may have compromised hip-ankle motor coordination mode, which can increase the risk of falls during activities of daily living. By investigating the effects of backpack weight on hip-ankle motor coordination mode, we can better design rehabilitation programs that improve postural stability and reduce the risk of falls.

## Further discussion

**Figure 13:**
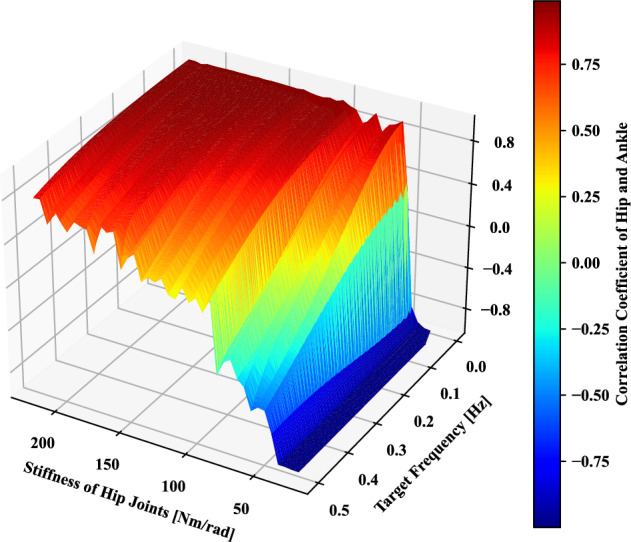
The correlation coefficient of hip and ankle joint angles in the whole tracking-balancing tasks is determined by the stiffness of hip joints from 25 to 200 [Nm/rad] and motion frequency *f* of the target point in Eq. (5) from 0.05 to 0.5 [Hz].

In this section, we discuss the influence of the combined impact factors of hip stiffness and tracking frequency on tracking performance and the switch of coordination patterns of the ankle and hip joints. As discussed above in the previous four experiments, mechanical and task-based constraints have a crucial impact on the self-organization between the ankle and hip joints. Thus, we would like to explore more cases by combining these constraints. Here, we take hip stiffness and tracking frequency as an example, considering mechanical and task-based constraints.

The primary purpose of this work is to explore the mechanism of emergent coordination modes between the ankle and hip joints. Here, the correlation coefficient of the hip and ankle joint angles is used to evaluate the coordination modes. Considering mechanical and task-based constraints for exploring the continuous transition between in-phase and anti-phase modes, two essential factors, including stiffness of hip joints and target tracking frequency that influence the postural coordination phase, are selected. In Fig. 13, with the increase of target tracking frequency and the decrease of stiffness of hip joints, the correlation coefficient changes from a positive value to a negative one means that postural coordination switches from in-phase to anti-phase. Mechanical constraints like hip stiffness can influence the phase modes as the upper body inertia effect appears more significantly for the reduced hip stiffness condition. In high hip stiffness, the postural coordination modes remain in-phase while the anti-phase is kept in low hip stiffness. For the hip stiffness ranging from 50 to 150 [Nm/rad], the coordination modes are heavily influenced by the target tracking frequency. Furthermore, the continuous transition from in-phase to anti-phase between the ankle and hip joint happened as the target tracking frequency increased in the condition of fixed hip stiffness, as discussed in the previous human experiments^17^.

We have also tested exploring the effect of head motion amplitude on mode alternation, but the alternation observed was not as clear as that of the frequency effect. As explained earlier, the acceleration and deceleration of mass play a crucial role in inducing mode alternation. According to mechanical dynamics, the amplitude itself has a relatively minor effect on this phenomenon. In the case of humans, the amplitude can have some impact, but our foot, being a rigid body on the floor, doesn’t provide much gripping capability. As a result, the sustainable amplitude range is not wide enough to observe a significant alternation for the amplitude test. To study this aspect further, we would require factors such as foot gripping function with foot fingers or friction.

## Conclusion and perspective

This study proposed and studied to produce a self-organizing neural network that can adaptively coordinate the postural mode without assuming a prior body model regarding body dynamics and kinematics. Postural coordination modes are reproduced in head-target tracking tasks through a deep reinforcement learning algorithm. Such a result is difficult to obtain with models based on offline optimization^21^ or the model-based control model^20^. The transitions between the postural coordination types, i.e. in-phase and anti-phase coordination modes, could be reproduced by changing frequencies of the moving target (Fig. 6). Mode alternation was consistent with observations reported in the literature^17,19^.

The correlation coefficient and relative phase indicated the two coordination modes, in-phase and anti-phase of the ankle and hip joints. These indices can indicate the spatial features of the ankle and hip joint angles. The emergence of postural modality was influenced by mechanical and task-based constraints with regard to hip stiffness, energy consumption, and frequency of tracking target. From the result of Fig. 8, we can conclude that energy penalization tends to change the ankle and hip joints’ coordination modes toward in-phase mode. From an energy-saving point of view, in-phase coordination consumes less energy than anti-phase coordination between the ankle and hip joints if there is a solution. However, the task involves a complex mass inertia effect, the hip joint gets required to be articulated intensively, then it turns into anti-phase mode. Body parameter like hip joint stiffness also could be confirmed to make influence the mode alternation. When the joint stiffness gets decreased, the necessity to compensate for the body inertia gets high, then anti-phase mode coordination is more required (Fig. 9).

Finally, the continuous transition between postural coordination modes was realized, it could demonstrate the learned neural network adaptability for a new task while keeping self-organized mode alternation. Even if the neural network is not trained under continuous frequency variations, it could adapt to the time-varying target frequency changes while keeping consistent mode alternation from in-phase to anti-phase. Further, expecting the learned neural network to manage the larger inertia effect, we have verified how the neural network may behave for the new task with an upper body mass increase. It is worth noting that the neural network is trained only with different target frequencies. For the increased upper body inertia, the mode was altered into anti-phase in a self-organizing manner.

This work provides a new approach to computational human motor learning and illustrates the postural modality of self-organization for head-tracking tasks. For future research, we may explore continuous coordination dynamics between the ankle and hip joints under other constraints or tasks such as body size and obesity, and functional reaching while standing. Furthermore, whole multi-segments, including neck, arm, hip, knee, and ankle joints, may be considered for body modeling to improve the current study toward full whole-body tasks. Finally, in-phase and anti-phase alternation is one of the synchronization phenomena in mechanical systems. More generalized perspective on synergetic synchronized oscillation is also an important future research direction to induce dynamic equilibrium in natural mechanical systems^41^.

## Supplementary Information

Supplementary Information 1.

Supplementary Information 2.

## Data Availability

All codes used to perform the analyses are available from the repository. https://github.com/Li-Guanda/SwingHumanoid.
