# Supplementary material for: Self-organizing neural network for reproducing human postural mode alternation through deep reinforcement learning
Source: Sci Rep. 2023 Jun 2;13:8966. doi: 10.1038/s41598-023-35886-y (PMC10238493; doi:10.1038/s41598-023-35886-y)
Supplement: Supplementary file 2 — Supplementary Information 2. [file 41598_2023_35886_MOESM2_ESM.pdf]

Supplementary materials

Video: HumanPosturalModeAlternation.MP4:

This video illustrates Posture Mode Alternation by Self-Organizing Neural Network.

A self-organized phenomenon in postural coordination is essential for understanding the auto-switching mechanism of in-phase and anti-phase postural coordination modes during standing and related supra-postural activities. Previously model-based approach was proposed to reproduce such self-organized phenomenon. However, if we set this problem including the process of how we establish the internal predictive model in our central nervous system, the learning process is critical to be considered for establishing a neural network for managing adaptive postural control. Particularly when body characteristics may change due to growth or aging or are initially unknown for infants, a learning capability can improve the hyper-adaptivity of human motor control for maintaining postural stability and saving energy in living.

This study attempted to generate a self-organizing neural network that can adaptively coordinate the postural mode without assuming a prior body model regarding body dynamics and kinematics. Postural coordination modes are reproduced in head-target tracking tasks through a deep reinforcement learning algorithm. The transitions between the postural coordination types, i.e. in-phase and anti-phase coordination modes, could be reproduced by changing the task condition of the head tracking target, by changing the frequencies of the moving target. These modes are considered emergent phenomena existing in human head tracking tasks. Various evaluation indices, such as correlation, and relative phase of hip and ankle joint, are analyzed to verify the self-organizing neural network performance to produce the postural coordination transition between the in-phase and anti-phase modes. In addition, after learning, the neural network can also adapt to continuous task condition changes and even to unlearned body mass conditions keeping consistent in-phase and anti-phase mode alternation.
